# Micro-consolidation occurs when learning an implicit motor sequence, but is not influenced by HIIT exercise

**DOI:** 10.1038/s41539-024-00238-6

**Published:** 2024-03-20

**Authors:** Emily Brooks, Sarah Wallis, Joshua Hendrikse, James Coxon

**Affiliations:** School of Psychological Sciences, Turner Institute for Brain and Mental Health, Monash University, Victoria, VIC 3800 Australia

**Keywords:** Consolidation, Motor control

## Abstract

We investigated if micro-consolidation, a phenomenon recently discovered during the brief rest periods between practice when learning an explicit motor sequence, generalises to learning an implicit motor sequence task. We demonstrate micro-consolidation occurs in the absence of explicit sequence awareness. We also investigated the effect of a preceding bout of high-intensity exercise, as exercise is known to augment the consolidation of new motor skills. Micro-consolidation was not modified by exercise.

Consolidation is a key learning process underpinning the development of motor skills. Consolidation refers to the strengthening of memory with increased resistance to interference, and can occur both online during periods of active practice, and offline between practice sessions^1^. Research typically focuses on consolidation occurring over a timescale of hours to days, but recent research has established that consolidation also occurs on a more rapid scale of seconds, termed micro-consolidation^2^. Bonstrup and colleagues^2^ utilised an explicit motor sequence task, alternating between task practice and rest every 10-seconds, to reveal that early performance gains were almost exclusively attributable to the brief rest periods^2,3^. This offline micro-consolidation may be underpinned by rapid neural replay of the sequence between the hippocampus and neocortical regions of the brain^4,5^, however, whether awareness of the sequence is required for micro-consolidation remains unclear.

In contrast to explicit sequence learning, implicit learning is characterised by behavioural improvement in the absence of conscious awareness of the sequence^6–8^, for example the increased speed and accuracy of key presses when using a keyboard over time. Implicit and explicit learning are thought to follow differing learning trajectories, and while there is some overlap, rely upon differing brain networks^6–12^. It is unclear whether the micro-consolidation effects reported for explicit sequence learning are also a feature of implicit motor sequence learning, with one study reporting micro-offline decrements^13^. Thus, our primary aim was to examine micro-consolidation when learning an implicit motor sequence task.

The consolidation of a novel motor skill can be enhanced by coupling task practice with an acute bout of high-intensity interval (HIIT) exercise^13–23^. However, studies to date have focused on offline consolidation over the timescale of hours to days, and it is unclear whether these effects are observed across shorter timescales. Therefore, as a secondary aim we investigated the priming effect of HIIT on micro-consolidation.

We hypothesised that early learning of an implicit motor sequence would be accounted for by performance gains during brief rest ‘micro-offline’ periods, and that a preceding bout of HIIT would enhance this micro-consolidation effect.

Thirty-eight (39.5% female) right-handed, healthy young adults aged 22.55 ± 2.69 (mean ± standard deviation; range 19–28) participated. The experiment involved a 20-min bout of either high-intensity cycling (HIIT group, *n* = 19) or very low intensity cycling (LOW group, *n* = 19), followed by the completion of an implicit serial reaction time task (SRTT)^8^, additional self-report measures, and determination of sequence awareness.

HIIT exercise involved cycling at alternating epochs of moderate intensity (3-min) and high intensity (2-min)^19,24^. The LOW group were required to turn the pedals at a very low cadence requiring minimal exertion.

The SRTT was completed approximately 30-min following exercise. Participants were instructed to respond as quickly and accurately as possible to visual cues, by pressing response buttons with the four fingers of their non-dominant left hand (Fig. 1). Undisclosed to participants, the visual cues followed a 12-item repeating sequence. A correct response was required to progress to the next cue. The task alternated every 10-s between task practice and rest for a total of 16 practice blocks, 320-s in total.

**Fig. 1:**
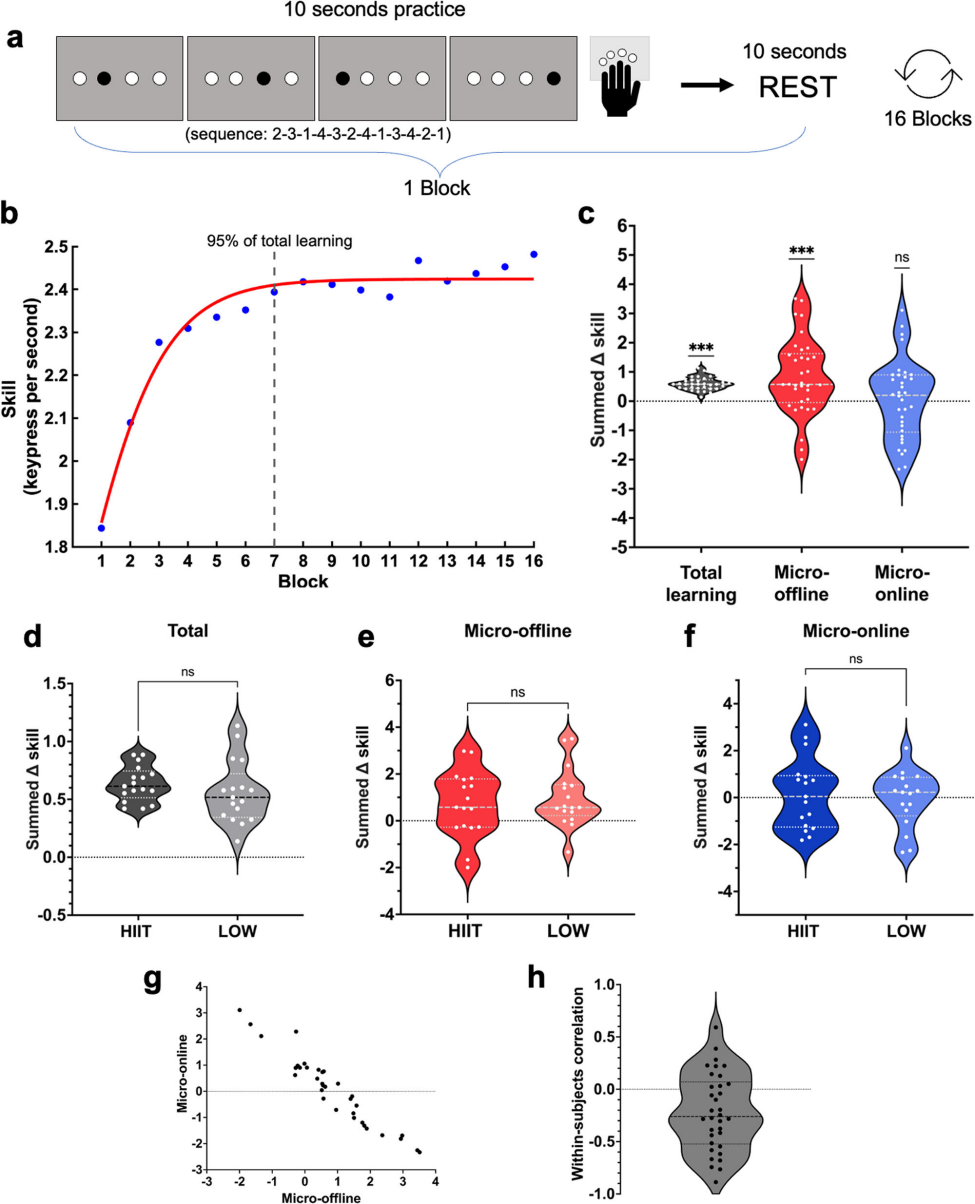
Implicit serial reaction time task paradigm and microscale learning effects. **a** The serial reaction time task (SRTT) protocol involved alternating between 10-seconds of practice and rest, for a total of 16 blocks. **b** SRTT performance across the session, representing the mean skill (key presses per second for correct responses) for all participants. The early learning period (i.e., in which 95% of total learning occurs) includes blocks 1–7. **c** Micro-consolidation of an implicit motor sequence. Total early learning (grey) on the SRTT was accounted for by micro-offline performance improvements (red). No significant improvements were seen during practice (blue). *ns* nonsignificant, ***p < 0.001. No significant differences were seen between HIIT and LOW groups for total learning (**d**), micro-offline consolidation (**e**) or micro-online consolidation (**f**), all *p* > 0.34, all BF_10_ < 0.47. **g** Between-subjects relationship between the micro-online and micro-offline effect, *r* = −0.96, *p* < 0.001. **h** Within-subjects relationship between the micro-online and micro-offline effect, group mean correlation coefficient of *r* = −0.22, SD .37, (t_33_ = −3.4, *p* = 0.002). For each participant the correlation coefficient between the first seven micro-online and micro-offline epochs was calculated.

As per Bonstrup and colleagues^2,3^, motor skill was conceptualised as number of correct responses per second, the learning trajectory was modelled at the group-level using an exponential function, and analysis was restricted to the ‘early learning’ phase, where 95% of learning occurred (i.e., blocks 1–7) (Fig. 1). Total early learning was defined as the overall summed change in skill. Micro-offline consolidation was the summed change in skill between blocks. Micro-online consolidation was the summed change in skill within blocks.

The HIIT and LOW groups were well matched (all *p* > 0.05) except for the expected difference in heart rate at the end of HIIT (*p* < 0.001) (Table 1). HIIT exercise was well-tolerated by participants.

**Table 1:** Comparison of participant characteristics between HIIT and LOW groups (Mean ± Standard Deviation)

	HIIT	LOW	*p* value
*N* (% Female)	19 (36.84%)	19 (42.11%)	0.75
Age (years)	22.37 ± 2.65	22.74 ± 2.79	0.68
BMI	22.06 ± 2.21	23.3 ± 4.14	0.26
IPAQ	4800 ± 3220	3783 ± 2739	0.30
PRETIE-Q T	26 ± 5.08	23.95 ± 4.92	0.21
PRETIE-Q P	26.32 ± 5.09	23.37 ± 4.22	0.06
PSQI	5.05 ± 2.53	4.16 ± 1.77	0.21
Resting HR	64.26 ± 9.75	65.37 ± 10.07	0.92
Exercise peak HR	180.68 ± 7.10	89.95 ± 10.96	<0.001

*BMI* Body Mass Index, *IPAQ* International Physical Activity Questionnaire (METS), higher values indicate greater physical activity levels, *PSQI* Pittsburgh Sleep Quality Index global score, with higher values indicating poorer sleep quality. *PRETIE-Q* Preference for and Tolerance of the Intensity of Exercise Questionnaire, higher exercise intensity tolerance (PRETIE-Q T) and preference (PRETIE-Q P) are indicated by higher values, *HR* Heart Rate in beats per minute. Resting HR was determined while seated. Exercise peak HR was obtained from the last minute of HIIT exercise.

Four participants (HIIT = 2, LOW = 2) were determined to have developed explicit awareness of the sequence (i.e., successfully reproduced five or more items from the 12-item sequence following task performance^25^) and were removed from the analysis. Across the entire sample we observed significant total learning (*M* = 0.60, *SD* = 0.22), t (33) = 15.89, *p* < 0.001, 95% CI [0.52, 0.68], *d* = 2.73. At the group level, this early learning was accounted for by performance gains during micro-offline periods (*M* = 0.84, *SD* = 1.32), t (33) = 3.72, *p* < 0.001, 95% CI [0.38, 1.30], *d* = 0.64, rather than micro-online periods (*M* = 0.05, *SD* = 1.35), t (33) = 0.22, *p* = 0.83, 95% CI [−0.42, 0.52] (Fig. 1). This finding extends on previous work that has investigated explicit sequence learning^2,3,5,26^. We demonstrate that early learning on an implicit motor sequence task occurs during brief ‘micro-offline’ rest periods, instead of during active practice.

Task accuracy was consistent across blocks (F(5.15,185.23) = 1.29, *p* = 0.27), with no group differences (HIIT: *M* = 0.98, *SD* = 0.006; LOW: *M* = 0. 96, *SD* = 0.01), F(1,36) = 1.35, *p* = 0.25). There were also no differences between the HIIT and LOW groups across primary outcome variables [total early learning (HIIT: *M* = 0.63, *SD* = 0.15, LOW: *M* = 0.56, *SD* = 0.27), t (32) = −0.96, *p* = 0.34, 95% CI [−0.23, 0.08], BF_10_ = 0.47; micro-online learning (HIIT: *M* = 0.72, *SD* = 1.41, LOW: *M* = 0. 97, *SD* = 1.25), t (32) = 0.55, *p* = .59, 95% CI [−0.68, 1.18], BF_10_ = 0.36; micro-offline learning (HIIT: *M* = 0.17, *SD* = 1.52, LOW: *M* = −0.07, *SD* = 1.20), t (32) = −0.49, *p* = 0.63, 95% CI [−1.19, 0.73], BF_10_ = 0.37 (Fig. 1)]. Overall, this finding indicates that a preceding bout of HIIT exercise did not influence micro-consolidation of an implicit motor sequence.

In summary, we investigated micro-consolidation during motor skill learning with an implicit serial reaction time task paradigm. In line with our hypothesis, we observed overall evidence of learning, with performance improvements at the group level accounted for by the brief ‘micro-offline’ rest periods between practice. We show that micro-consolidation occurs in the context of an implicit motor sequence paradigm. This finding contrasts with Fanuel and colleagues^13^ which may be attributable to differences in task demands and structure (i.e., the probabilistic nature of their task and the variable duration of rest and practice periods). Our results indicate that the micro-consolidation phenomenon extends to motor skill learning of an implicit sequence. A strength of our study is that we closely matched features of the original studies reporting micro-consolidation by Bonstrup and colleagues^2,3^ namely the alternating 10 s task structure, the data analysis methods, and operationalisation of outcome measures. Overall, our results add to a growing body of evidence^2,3,5,26^ indicating that motor skills are consolidated rapidly across a scale of seconds, with improvements developing during brief periods of rest. Our results indicate that explicit sequence awareness is not required for micro-consolidation.

Previous lines of evidence have implicated hippocampal declarative networks in explicit sequence learning^8,11^, with hippocampal-neocortical replay a likely mechanism contributing to learning of novel motor skills^4,5^ and micro-consolidation of explicit sequences^2^. In contrast, implicit sequence learning is supported by cortico-striatal networks involved in procedural memory formation^8^. Our results implicate micro-consolidation as a process shared across memory systems with distinct topology. Future research is required to elucidate the neural mechanisms of implicit sequence micro-consolidation and whether there is overlap with explicit micro-consolidation. We acknowledge that the serial reaction task utilised in the current study featured a 12-item repeating sequence, which differs from both the seminal micro-consolidation work utilising an explicit 5-item sequence and other versions of the serial reaction time task which typically include comparison of the fixed and random sequences to quantify the magnitude of implicit learning. Further, although we observed robust effects at the group level, it is notable that there was a relationship between the magnitude of online and offline gains across participants. The reasons for this are unclear and will require further investigation. As outlined in our supplementary analyses (Supplementary Figs. 1–4), we observe some evidence of different response patterns between early and late stages of each iteration of the implicit motor sequence. It is plausible that these response dynamics reflect variables not measured herein, such as fatigue/attentional drift, reactive interference, or certain neuromusculoskeletal/biomechanical constraints imposed by the motor sequence. Alternatively, these response dynamics may indeed reflect skill learning and offline micro-consolidation. Beyond the behavioural analyses we have presented, future work is required to understand the neurophysiological mechanisms mediating these effects.

Contrary to our expectations, a preceding bout of HIIT did not influence micro-consolidation of motor skill learning for our task. Previous work has demonstrated minimal effects of HIIT on total learning during acquisition, but enhanced consolidation in the hours to days following practice^13–23^. The temporal dynamics of combining exercise and motor learning are not fully understood, but our results indicate that HIIT is unlikely to impact consolidation on the micro timescale when performed in close temporal proximity to the task.

In summary, we show that micro-consolidation occurs during motor sequence learning with an implicit serial reaction time task paradigm, suggesting that micro-consolidation is a general feature of early sequence learning that does not necessitate explicit awareness.

## Methods

### Experimental design

Participants were pseudo-randomly allocated to either the HIIT exercise or LOW condition while minimising between-group variance for age and biological sex. Participants completed an experimental session which involved a series of self-report questionnaires, a 20-min bout of exercise, and the serial reaction time task (SRTT). This study was approved by the Monash University Human Research Ethics Committee (MUHREC 27742), and all participants provided written informed consent prior to participating.

### Participant screening

Participants were screened using the adult pre-exercise screening tool^27^, and were required to be right handed, have no contraindications to exercise (e.g., physical injury, asthma, blood pressure problems, family history of heart disease), no history of neurological illness or injury, and no current prescriptions of psychoactive medication. Handedness was determined by the Edinburgh Handedness Inventory^28^ (EHI). All participants were right hand dominant for writing (*M* = 90.42, *SD* = 23.72).’

### Exercise protocol

Participants completed a 20-min bout of exercise on a cycle ergometer, with the intensity tailored to each individual based on their heart rate reserve (HRR) (maximum heart rate (MHR) = 220 – age, HRR = MHR – resting heart rate (RHR)). The HIIT protocol was conducted as per previous studies^19,24,29,30^, and involved alternating between 3 min of moderate intensity (target heart rate of 50–60% HRR) and 2 min of high intensity cycling (target heart rate of up to 90% HRR), while the LOW group were required to cycle at a very low cadence with minimal exertion (the target heart rate below 20% HRR). Participant’s rating of perceived exertion (RPE) was recorded each minute using the original version of the BORG scale, where ratings range from 6 (no exertion) to 20 (maximal exertion)^31^.

### SRTT

Participants were provided with a button box with four buttons, and mapped the fingers of their left hand such that their little finger aligned to the left most button and index finger to the right most button. On each trial, participants were presented with four white circles arranged horizontally on a monitor screen and were instructed to respond to the circle that turned black by pressing the corresponding button as quickly and accurately as possible. Undisclosed to participants, the visual cues followed a 12-item repeating sequence (2-3-1-4-3-2-4-1-3-4-2-1). A correct response was required to progress to the next trial, and the task alternated every 10-seconds between task practice and rest for a total of 16 practice blocks, 320-s in total. Following completion of the task, participants were questioned about their level of explicit sequence awareness. Participants who were able to accurately replicate the first five or more sequence items were determined to have gained some degree of explicit sequence awareness (*N* = 4; HIIT = 2, LOW = 2), and were removed from subsequent analyses.

### Statistical analysis

As per previous studies^2,3^, motor skill performance was conceptualised as number of correct responses per second, the learning trajectory was modelled at the group-level using an exponential function, and analysis was restricted to the ‘early learning’ phase, where 95% of learning occurred (i.e., blocks 1–7). ‘To examine procedural motor-skill learning within and between blocks, a sliding average with a window of four-items was used. The average tapping speed (quantified as keypresses/s) across the first and last four correct key presses in each block represented performance at the start and end of each block, respectively. Micro-offline was the summed change between blocks, i.e., the summed change in tapping speed between the start of block *n* + 1 and the end of block n. Micro-online was the summed change within blocks, i.e., the summed change in tapping speed between start and end of block n. Total early learning was calculated by summing micro-online and micro-offline values.

All data passed assumption checks of normality and homogeneity of variance. Three one sample t-tests were conducted across the whole sample to assess changes in micro-online, micro-offline, and total early learning. Three independent samples *t*-tests were conducted to investigate differences in micro-online, micro-offline, and total early learning between HIIT and LOW groups. Three Bayesian independent samples *t*-tests (priors set in support of the alternative over the null hypothesis, i.e., BF_10_, and the Cauchy set to the conservative default 0.707) were also conducted to quantify the relative evidence for the alternative vs. the null hypothesis across micro-online, micro-offline, and total early learning variables between HIIT and LOW groups.

### Supplementary analyses

To assess the possibility that microscale effects in blocks 1–7 were influenced by a primacy bias to initial cues (i.e., systematically faster responses to initial cues in the implicit motor sequence), we conducted supplementary analyses of each subject’s response times across completion of the first instance of the 12-item sequence (i.e, first 12 key presses) for the first four, middle four, and last four key presses of this sequence.

To test the possibility of generalised slowing across keypresses within each 10 s task epoch, a linear regression model (polyfit function, matlab) was fit to each subject’s intertap intervals. The regression model slope parameter was extracted from the model. For this analysis, a positive slope value would be indicative of a progressive slowing of response times across a task epoch. We also conducted analyses omitting the first block. We compared correct key presses (per second) for trials 2–5 and 7–10 of the sequence, and averaged across blocks 4–7 for each participant.

### Reporting summary

Further information on research design is available in the Nature Research Reporting Summary linked to this article.

### Supplementary information

Supplementary Materials

Reporting summary

## Data Availability

De-identified behavioural data are available at [https://osf.io/4e3xr/].

## Code availability

All code used for analysis is available at [https://github.com/jhendrikse/micro_consolidation].
