# Supplementary material for: Micro-consolidation occurs when learning an implicit motor sequence, but is not influenced by HIIT exercise
Source: NPJ Sci Learn. 2024 Mar 20;9:23. doi: 10.1038/s41539-024-00238-6 (PMC10954609; doi:10.1038/s41539-024-00238-6)
Supplement: Supplementary file 1 — Supplementary Materials [file 41539_2024_238_MOESM1_ESM.pdf]

Supplementary materials

Individual learning variability

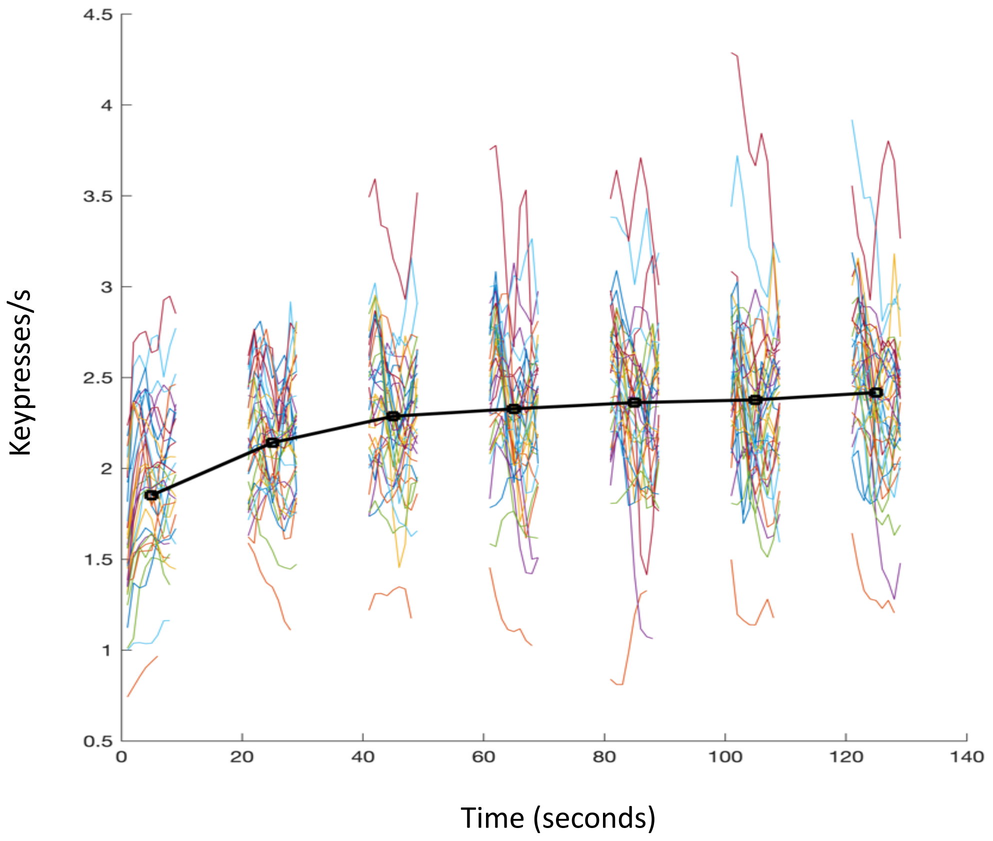

Supplementary Figure 1. Individual change in keypresses/s across the early learning period (blocks 1-7) (represented in seconds). Group mean performance is superimposed in black.

Assessment of primacy response bias

Repeated measures ANOVA, within factors of Block (i.e., block 1-7) and Response Epoch (i.e., first four, middle four, last four key presses) revealed a significant Block x Response Epoch interaction ($F = 22.61$, $p < .001$, $\eta_p^2 = 0.42$). However, visual inspection of the data (see figures below) and post-hoc tests show that this interaction was attributable to significantly *slower* responses to the first four initial cues in block 1 (first four presses, block 1 vs mid four presses, block 1, mean difference = 0.21, $p < .001$, Cohen's d = 2.43; first four presses, block 1 vs last four presses block 1, mean difference = 0.18, $p < .001$, Cohen's d = 2.05). Although we observed slightly faster response times for early keypresses in blocks 4-7, post-hoc comparisons revealed no significant differences for early versus mid, and late epochs of the sequence (all $p > .11$).

Overall, the results of this supplementary analysis do not provide evidence to support the notion that microscale improvements are driven by a primacy effect. Instead, our findings of a micro-offline effect in the absence of clear primacy effects supports the notion of microscale sequence consolidation.

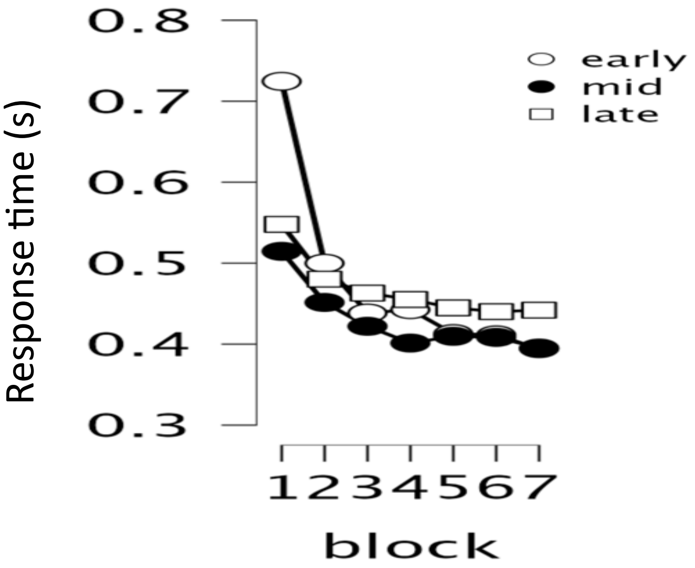

Supplementary Figure 2A. Significantly slower reaction times to the first four cues, relative to the middle and last four cues in block 1.

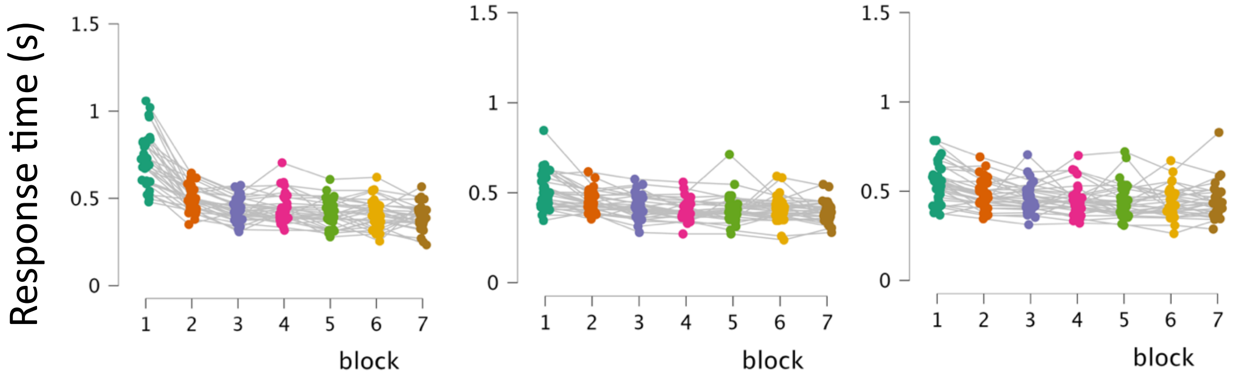

Supplementary Figure 2B. Individualised data – evidence of significantly slower reaction times to the first four cues in block 1 (left), relative to the middle (middle panel) and last four cues (right).

Assessment of fatigability

A repeated measures ANOVA on the slope parameter values (within factor of Block 1-7) revealed a significant main effect of block (F = 10.90, p < .001, partial eta squared = 0.254). However, post-hoc tests (one sample t-tests), indicated that this effect was primarily attributable to a significant *negative* slope across block one (p < .001, Cohen's d = 0.68) (i.e., evidence of faster intertap intervals across the keypresses comprising the first task epoch, see figure below). We note the distribution of slope parameters in block 7 is positive (p = .004, Cohen's d =0.53), but this effect is influenced by one outlier value.

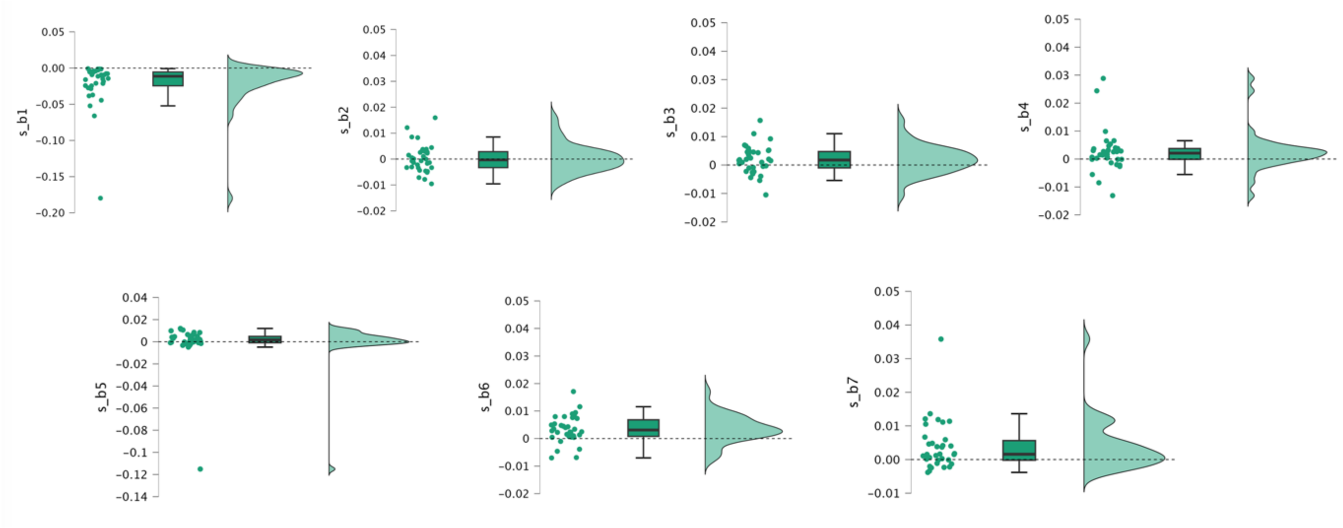

Supplementary Figure 3. Comparison of slope parameter estimates for each participant across blocks (s_b1 -s_b7) – negative slope estimates in block 1 indicate progressively faster response times across the keypresses of the first task epoch.

Notably, these slope parameters were not correlated with micro-online (all p > .20) or micro-offline (all p > .28) effects.

Further analysis of performance within blocks

We observed some indication that responses differed across segments of the sequence, significant one-sample t-test (p < .001).

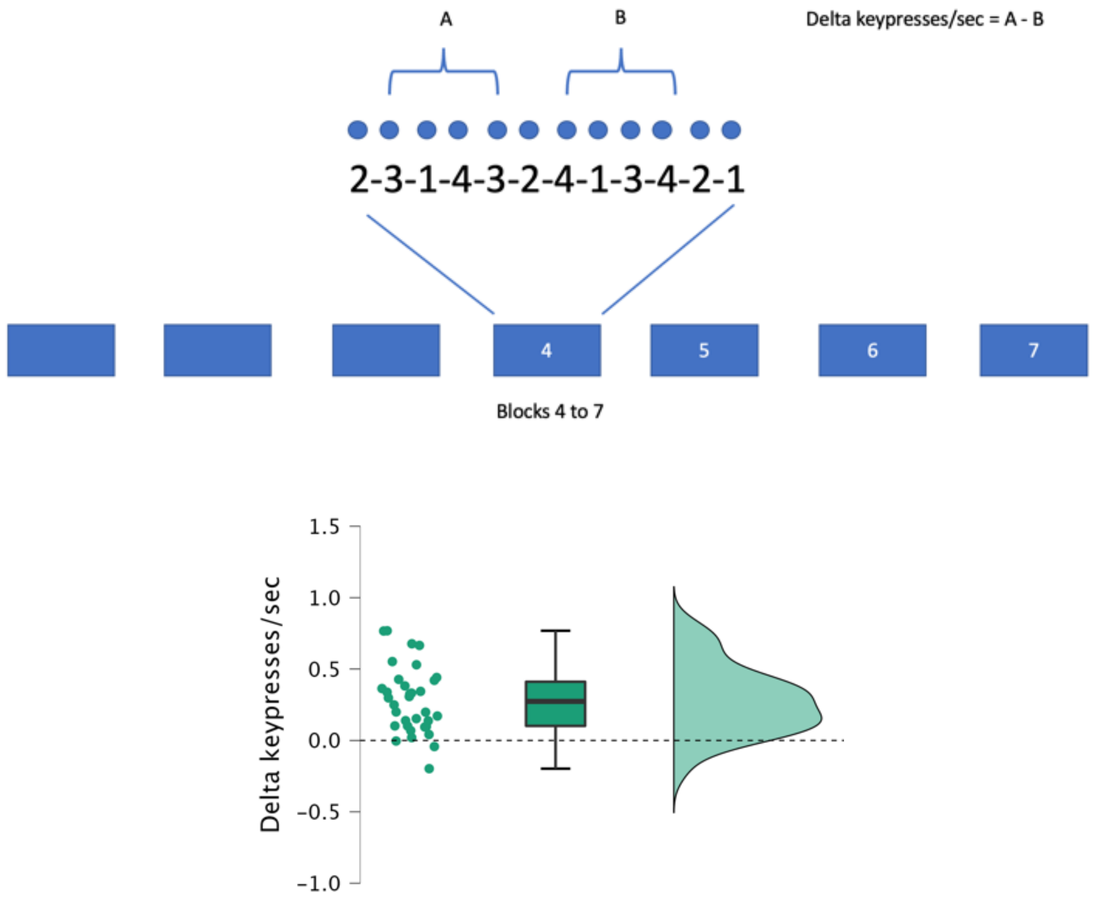

Supplementary Figure 4. Top: Correct key presses (per second) for trials 2-5 (i.e A) and 7-10 (i.e B) of the sequence were compared (A-B) to give delta keypresses/sec, and then averaged across blocks 4-7 for each participant. Bottom: One sample t-test for the delta keypresses/sec for each participant ($t_{33} = 6.93$, $p < .001$).

The results of this analysis are consistent with analyses presented above. As with any behavioural study, the mechanistic underpinnings cannot be known for certain. Future research using an implicit serial reaction time task combined with neural measures of micro-consolidation (e.g. Buch et al, 2020) may be informative.
