# Supplementary material for: Micro-consolidation occurs when learning an implicit motor sequence, but is not influenced by HIIT exercise
Source: NPJ Sci Learn. 2024 Mar 20;9:23. doi: 10.1038/s41539-024-00238-6 (PMC10954609; doi:10.1038/s41539-024-00238-6)
Supplement: Supplementary file 2 — Reporting summary [file 41539_2024_238_MOESM2_ESM.pdf]

nature portfolio

Corresponding author(s): James Coxon

Last updated by author(s): 2023.09.02

Reporting Summary

Nature Portfolio wishes to improve the reproducibility of the work that we publish. This form provides structure for consistency and transparency in reporting. For further information on Nature Portfolio policies, see our Editorial Policies and the Editorial Policy Checklist.

Statistics

For all statistical analyses, confirm that the following items are present in the figure legend, table legend, main text, or Methods section.

n/a	Confirmed	
☐	☒	The exact sample size (*n*) for each experimental group/condition, given as a discrete number and unit of measurement
☐	☒	A statement on whether measurements were taken from distinct samples or whether the same sample was measured repeatedly
☐	☒	The statistical test(s) used AND whether they are one- or two-sided<br>*Only common tests should be described solely by name; describe more complex techniques in the Methods section.*
☐	☒	A description of all covariates tested
☐	☒	A description of any assumptions or corrections, such as tests of normality and adjustment for multiple comparisons
☐	☒	A full description of the statistical parameters including central tendency (e.g. means) or other basic estimates (e.g. regression coefficient) AND variation (e.g. standard deviation) or associated estimates of uncertainty (e.g. confidence intervals)
☐	☒	For null hypothesis testing, the test statistic (e.g. *F*, *t*, *r*) with confidence intervals, effect sizes, degrees of freedom and *P* value noted<br>*Give P values as exact values whenever suitable.*
☐	☒	For Bayesian analysis, information on the choice of priors and Markov chain Monte Carlo settings
☐	☒	For hierarchical and complex designs, identification of the appropriate level for tests and full reporting of outcomes
☐	☒	Estimates of effect sizes (e.g. Cohen's *d*, Pearson's *r*), indicating how they were calculated

Our web collection on statistics for biologists contains articles on many of the points above.

Software and code

Policy information about availability of computer code

Data collection	Custom PsycoPy code was used to collect the motor task data
Data analysis	Custom Matlab scripts were used and are available at [https://github.com/jhendrikse/].

For manuscripts utilizing custom algorithms or software that are central to the research but not yet described in published literature, software must be made available to editors and reviewers. We strongly encourage code deposition in a community repository (e.g. GitHub). See the Nature Portfolio guidelines for submitting code & software for further information.

Data

Policy information about availability of data

All manuscripts must include a data availability statement. This statement should provide the following information, where applicable:

- Accession codes, unique identifiers, or web links for publicly available datasets
- A description of any restrictions on data availability
- For clinical datasets or third party data, please ensure that the statement adheres to our policy

De-identified behavioural data are available at [https://osf.io/4e3xr/], and all code used for analysis is available at [https://github.com/jhendrikse/].

Research involving human participants, their data, or biological material

Policy information about studies with human participants or human data. See also policy information about sex, gender (identity/presentation), and sexual orientation and race, ethnicity and racism.

Reporting on sex and gender	Biological sex was recorded, but not investigated beyond sample characteristics.
Reporting on race, ethnicity, or other socially relevant groupings	This information was not recorded as it was not relevant to current research question.
Population characteristics	see below
Recruitment	Participants were recruited via posters around the Monash University campus, advertisements on social media and word of mouth snowballing
Ethics oversight	Monash University Human Research Ethics Committee (MUHREC 27742)

Note that full information on the approval of the study protocol must also be provided in the manuscript.

Field-specific reporting

Please select the one below that is the best fit for your research. If you are not sure, read the appropriate sections before making your selection.

☐ Life sciences ☒ Behavioural & social sciences ☐ Ecological, evolutionary & environmental sciences

For a reference copy of the document with all sections, see nature.com/documents/nr-reporting-summary-flat.pdf

Behavioural & social sciences study design

All studies must disclose on these points even when the disclosure is negative.

Study description	quantitative experimental, between subjects design
Research sample	Right-handed, healthy young adults (aged 18-35) participated. Participants were screened using the adult pre-exercise screening tool, and were required to be right handed, have no contraindications to exercise (e.g., physical injury, asthma, blood pressure problems, family history of heart disease), no history of neurological illness or injury, and no current prescriptions of psychoactive medication.
Sampling strategy	Random sampling was used. A power analysis indicated a sample size of 38 would be sufficient..
Data collection	Surveys were completed electronically via a computer or ipad. Exercise data was recorded electronically via Wattbike. A computer and response box was used to complete the task and record responses. Researchers supervised the exercise and were therefore not blinded to experimental condition.
Timing	Data collection occurred from March - August 2022
Data exclusions	Participants who were able to accurately replicate the first five or more sequence items were determined to have gained some degree of explicit sequence awareness (N = 4; HIIT = 2, LOW = 2), and were removed from subsequent analyses.
Non-participation	No participants withdrew from the study
Randomization	Participants were pseudo-randomly allocated (via Rando.la) to either the HIIT exercise or LOW condition while minimising between-group variance for age and biological sex

Reporting for specific materials, systems and methods

We require information from authors about some types of materials, experimental systems and methods used in many studies. Here, indicate whether each material, system or method listed is relevant to your study. If you are not sure if a list item applies to your research, read the appropriate section before selecting a response.

Materials & experimental systems

n/a	Involved in the study
☒	☐ Antibodies
☒	☐ Eukaryotic cell lines
☒	☐ Palaeontology and archaeology
☒	☐ Animals and other organisms
☒	☐ Clinical data
☒	☐ Dual use research of concern
☒	☐ Plants

Methods

n/a	Involved in the study
☒	☐ ChIP-seq
☒	☐ Flow cytometry
☒	☐ MRI-based neuroimaging
